# Potentially Pathogenic *Vibrio* spp. in Algal Wrack Accumulations on Baltic Sea Sandy Beaches

**DOI:** 10.3390/microorganisms12102101

**Published:** 2024-10-21

**Authors:** Marija Kataržytė, Greta Gyraitė, Greta Kalvaitienė, Diana Vaičiūtė, Otilija Budrytė, Martynas Bučas

**Affiliations:** 1 Marine Research Institute, Klaipėda University, University Avenue 17, 92295 Klaipėda, Lithuania; greta.gyraite@ku.lt (G.G.); greta.kalvaitiene@ku.lt (G.K.); diana.vaiciute@ku.lt (D.V.); martynas.bucas@ku.lt (M.B.); 2 Institute of Agriculture, Lithuanian Research Centre for Agriculture and Forestry, Instituto al. 1, Akademija, 58344 Kėdainiai, Lithuania

**Keywords:** algae wrack, recreational waters, *Vibrio vulnificus*, *Vibrio cholerae*, One Health

## Abstract

The *Vibrio* bacteria known to cause infections to humans and wildlife have been largely overlooked in coastal environments affected by beach wrack accumulations from seaweed or seagrasses. This study presents findings on the presence and distribution of potentially pathogenic *Vibrio* species on coastal beaches that are used for recreation and are affected by red-algae-dominated wrack. Using species-specific primers and 16S rRNA gene amplicon sequencing, we identified *V. vulnificus*, *V*. *cholerae* (non-toxigenic), and *V*. *alginolyticus*, along with 14 operational taxonomic units (OTUs) belonging to the *Vibrio* genus in such an environment. *V*. *vulnificus* and *V*. *cholerae* were most frequently found in water at wrack accumulation sites and within the wrack itself compared to sites without wrack. Several OTUs were exclusive to wrack accumulation sites. For the abundance and presence of *V*. *vulnificus* and the presence of *V. cholerae*, the most important factors in the water were the proportion of *V. fucoides* in the wrack, chl-a, and CDOM. Specific *Vibrio* OTUs correlated with salinity, water temperature, cryptophyte, and blue-green algae concentrations. To better understand the role of wrack accumulations in *Vibrio* abundance and community composition, future research should include different degradation stages of wrack, evaluate the link with nutrient release, and investigate microbial food-web interactions within such ecosystems, focusing on potentially pathogenic *Vibrio* species that could be harmful both for humans and wildlife.

## 1. Introduction

The Bathing Water Directive (BWD), which regulates bathing water quality monitoring in the European Union (EU) [1], includes only thresholds for *Escherichia coli* and *Enterococcus* spp., which are associated with fecal pollution risks. However, other microorganisms unrelated to fecal pollution can simultaneously be found in recreational coastal bathing areas, such as *Vibrio* bacteria [2]. Unlike fecal indicator bacteria, *Vibrio* spp. are common autochthonous bacteria in water. Several *Vibrio* species are potentially pathogenic and widely distributed across the globe [3].

*Vibrio*-related infections are increasing worldwide in humans and aquatic animals. This rise has been associated with the global increase in sea surface temperatures, the primary physical consequence of global warming [4]. *Vibrio* infections, associated with heat waves, have also been observed in the Baltic Sea in the last decade [5,6,7].

Several *Vibrio* species are considered of concern in the Baltic Sea: *V. alginolyticus*, *V. parahaemolyticus, V. vulnificus,* and *V. cholerae* [7]. Serogroups designated as *V*. *cholerae* non-O1/non-O139 are prevalent in the Baltic Sea and might cause wound or ear infections and gastroenteritis. *V. vulnificus* can lead to severe wound infections, particularly in immunocompromised people [3]. Given the low salinity and highly eutrophied water conditions typical for the Baltic Sea, which are preferred by pathogenic *Vibrio* bacteria [8,9], it is crucial to consider the trend of rapidly increasing sea surface temperature in this area since these conditions are expected to become more favorable for these pathogens’ growth [6,7,10].

*Vibrio* spp. can be considered an essential constituent of the macrophyte microbiome [11,12,13], contributing to the biogeochemical cycling of nutrients and potentially controlling pathogens in seawater [14]. Considering that the surface of live macrophytes can be a reservoir for *V. cholerae* [15] and *V. vulnificus* [16], after detachment and by accumulating on the coastal beaches as wrack, they also might serve as a reservoir for these *Vibrio* species. Beach wrack, an accumulation of macroalgae or seagrasses, including microalgae, animal carcasses, shells, wood, and higher plant debris, has an important ecological value for coastal ecosystems. In the western part of the Baltic Sea, wrack is primarily dominated by angiosperms, while the eastern part is dominated by accumulations of red and brown algae (*Rhodophyceae* and *Phaeophyceae*) [17,18]. Wrack might also play a role in the entanglement of plastic, which is known to act as a vector for transporting *Vibrio* bacteria [19]. *Vibrio* bacteria can form biofilms on the surfaces of plastic debris, thus potentially increasing their persistence and acting as the source of potential pathogens and horizontal gene transfer [20]. Moreover, wrack itself can support the survival of fecal bacteria [13,21,22] and potentially pathogenic microorganisms such as *Shigella*, *Salmonella*, and *Campylobacter* [23] due to the release of dissolved organic compounds into the aquatic environment [24], its surface for forming biofilms, and protection from harmful UV light and predation [25]. However, research on *Vibrio* bacteria in beach wrack worldwide is scarce or nonexistent [13].

Considering the abovementioned aspects, we aimed to assess the presence, abundance, and diversity of potentially pathogenic *Vibrio* bacteria in the recreational sandy beach areas of the southeastern Baltic Sea coast and the relation with wrack accumulation. This knowledge might have important implications for safeguarding public health on coastal beaches.

## 2. Materials and Methods

### 2.1. Study Area and Sampling Strategy

**Figure 1:**
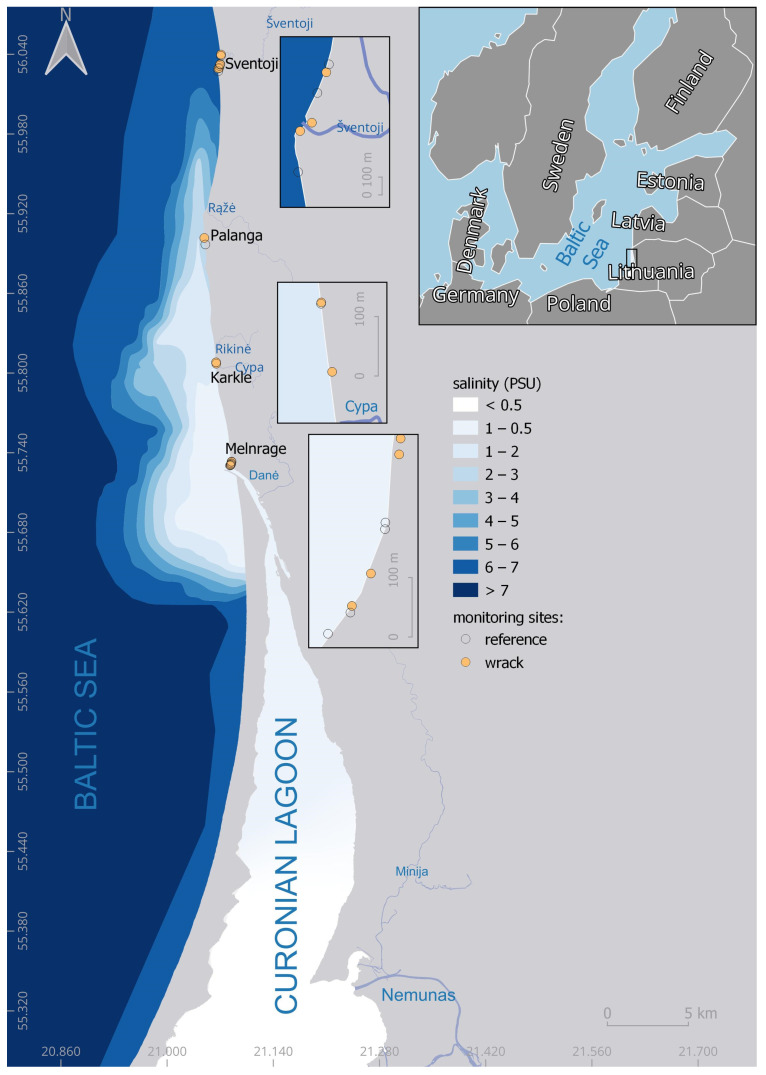
Sampling sites on Lithuania’s Baltic Sea coast along the salinity gradient. The map was created using salinity data from August 2022 from the Copernicus Marine Service product [26].

Samples were taken along the Lithuanian Baltic Sea coastline on four beaches—Melnragė, Karklė, Palanga, and Šventoji, covering the coastal stretch from the Curonian Lagoon outflow toward the Latvian coast with a salinity gradient affected by the lagoon outflow (Figure 1).

Environmental samples (wrack, water, and sand) were collected in 2021 (in June, July, August, and September) based on beach wrack accumulation events (nine sampling campaigns). In 2022, a multi-day sampling campaign was performed in Šventoji (one sampling event). Sampling campaigns were performed during the bathing season.

Two subsites were selected for each sampling campaign—an area with accumulated wrack and that without (reference site). Three separate samples of water, sand, and/or wrack (only from the wrack site) were collected from each site at places at a distance of 1 m (more in [21]). Before DNA extraction, replicates of each sample were pooled to reduce the costs [27]. Besides environmental samples, plastic items (n = 30) were collected from sites with wrack accumulation (n = 21) and sand in sites without accumulations (n = 9) [28].

Water was collected using sterile 200 mL Nalgene bottles to assess environmental parameters. Sand samples were gathered in conical 50 mL tubes (VWR), and wrack samples were placed in Whirl-Pak bags (VWR). All samples were immediately stored in a cooling box, transported to the laboratory, and processed within four hours. Environmental parameters, including chlorophyll-a (chl-a) (mg/m^3^) and phycocyanin (mg/m^3^), were measured on-site using an AlgaeTorch (bbe Moldaenke GmbH, Schwentinental, Germany)—a fluorescence measurement device. Turbidity (NTU), temperature (°C), oxygen (mg/L), salinity (PSU), and pH were measured on-site with a YSI Professional Plus Environmental multimeter probe (Xylem Analytics, Yellow Springs, OH, USA) (more in [21]).

### 2.2. Sample Processing in the Laboratory

Water samples were analyzed in the laboratory for chl-a and colored dissolved organic matter (aCDOM) using spectrophotometry [29,30,31]. Suspended particulate matter (SPM) was analyzed gravimetrically [32], and organic and inorganic fractions were determined after filters were combusted at 550 °C for four hours. Detailed information on how environmental parameters were analyzed is provided in [21,22].

Sand and wrack samples were diluted with 110 mL of sterile MiliQ water and ultrasonicated for 15 s with 30 s breaks, with an intensity of 1 W/cm^2^. This was repeated eight times using an ultrasonic bath (Bandelin Sonorex Digiplus, Berlin, Germany) to detach bacterial biofilm from sand and macrophytes.

For molecular analysis, water was filtered (Advantec Membrane Filter, Toyo Roshi Kaisha, Ltd., Tokyo, Japan) and kept in a −80 °C freezer before DNA extraction. Genomic DNA was extracted using DNeasy^®^ PowerWater^®^ Kit (QIAGEN, Hilden, Germany) and kept in a −20 °C freezer for further molecular analysis.

The dry weight of wrack samples was assessed by drying (at 60 °C) the samples until a constant weight. The algal species were identified using a Nikon SMZ800N stereomicroscope (Nikon, Tokyo, Japan). The species composition of the macrophyte community in the wrack, along with the environmental parameter data, is provided in [21].

### 2.3. Identification and Quantification of Vibrio Bacteria Using Molecular Methods

Four species of potentially pathogenic bacteria belonging to the *Vibrio* genus were targeted in DNA samples (both environmental and plastic)—*V. vulnificus*, *V. cholerae*, *V. parahaemolyticus*, and *V. alginolyticus*. Conventional PCR was used to identify the presence of these potential pathogens using species-specific primers in the water, sediment, and wrack samples (Supplementary Table S1).

Reaction mixtures for PCR contained 12.5 μL Platinum™ Green Hot Start PCR Master Mix (2X) (Invitrogen, Thermo Fisher Scientific, Carlsbad, CA, USA), 7.5 μL water for molecular biology (Sigma-Aldrich by Merck, Merck KGaA, Darmstadt, Germany), 0.2 μM of each primer, 3 μL of DNA. The final volume of the reaction mixture was 25 µL. The PCR reaction was performed in a ProFlex PCR thermocycler (Thermo Fisher Scientific, Waltham, MA, USA); the conditions for the amplification were as follows: denaturation of 1 min at 94 °C, followed by 30 cycles of denaturation at 94 °C for 1 min, primer annealing at 53 °C for 1 min, and extension at 72 °C for 1.5 min, and final extension at 72 °C for 10 min [33].

PCR products were visualized after electrophoresis on a 1.5% agarose gel, stained with SYBR^®^ Safe DNA Gel stain (Invitrogen, Thermo Fisher Scientific, Invitrogen, Waltham, MA, USA), and observed under UV light. *Vibrio vulnificus* was quantified using TaqMan-based qPCR assays. Standard curves were generated from triplicate samples of 10-fold serial dilutions of purified *V. vulnificus* DNA, with concentrations ranging from 10^8^ to 10^3^ gene copies.

Each sample was analyzed using TaqMan Universal PCR Master Mix (Applied Biosystems by Thermo Fisher Scientific, Foster City, CA, USA). Each qPCR run included a positive control, a negative extraction, and a non-template control. qPCR amplification was performed using the StepOnePlus^TM^ real-time PCR system (Applied Biosystems by Thermo Fisher Scientific).

The extracted DNA samples were also shipped for Illumina NovaSeq 6000 16s rRNA gene amplicon sequencing at Novogen Inc., Cambridge, UK. The forward (CCTAYGGGRBGCASCAG) and reverse (GGACTACNNGGGTATCTAAT) primers were used to target the V3-V4 hypervariable regions of the 16S rRNA gene. Sequencing libraries were prepared using the TruSeq^®^ DNA PCR-Free Sample Preparation Kit (Illumina, USA). Paired-end reads were merged using FLASH (V1.2.11, http://ccb.jhu.edu/software/FLASH/, accessed on 15 June 2023). Data Filtration Quality filtering on the raw tags was performed using fastp (Version 0.23.1) software to obtain high-quality clean tags [34]. The tags were compared with the reference database (Silva database (16S), https://www.arb-silva.de/, accessed on 15 June 2023) using the UCHIME algorithm to detect chimera sequences, and then the chimera sequences were removed [35]. Sequence analyses were performed by Uparse software (Uparse v7.0. 1001, http://drive5.com/uparse/, accessed on 15 June 2023). Sequences with ≥97% similarity were assigned to the same operational taxonomic unit (OTU). Taxonomic information for each representative sequence was annotated using the Silva Database (http://www.arb-silva.de/, accessed on 10 August 2024) [36], based on the Mothur algorithm. The representative sequences of *Vibrio* OTUs were further identified by a blast search against the NCBI database (https://blast.ncbi.nlm.nih.gov/Blast.cgi, accessed on 16 August 2024). The sequence data were uploaded to NCBI BioProject under the accession number PRJNA1067868.

Phylogenetic analysis was performed by using neighbor-joining in mega 11 [37]. Bootstrap analysis [38] was performed for 1000 replications.

### 2.4. Data and Statistical Analysis

A systematic literature review was performed to discover similar studies that analyzed *Vibrio* presence in beach wrack worldwide, both in freshwater and marine environments. We selected several databases to search for publications: Google Scholar, PubMed, Web of Science (WoS), and Scopus. A search was performed by combining several keywords with Boolean operators “AND” and “OR”: “Vibrio” AND “cast” OR “wrack” OR “beachcast” OR “debris” OR “detritus”. The search was performed on 26 August 2024 and resulted in only one publication that analyzed *Vibrio* bacteria presence in beach wrack in the Mediterranean. Information extracted from this publication is provided in Supplementary Table S6.

Statistical tests were performed using the R software (version 4.4.1) environment (R Core Team, 2023). Figures were composed using the ‘ggplot2’ (3.4.4) package [39] in R software and Microsoft Excel 2019.

Spearman correlation was used to estimate the strength and significance of the relationships between *V. vulnificus* abundance and environmental parameters in water and OTUs’ relative abundance and environmental parameters in water.

The Kruskal–Wallis test was used to assess differences between environmental parameters. Comparisons between categorical variables (presence/absence) were made using the chi-square test. The importance of environmental factors in explaining the variation in the abundance and presence of *V. vulnificus* and the presence of *V. cholerae* was assessed using a multivariate Random Forest regression model (MRF), due to the relatively small dataset and high multicollinearity among the explanatory variables; e.g., chl-a was highly (r > 0.8) correlated with CDOM and *V*. *fucoides*. The MRF was performed using the “randomForestSRC” package [40] in R. The number of trees (250) was selected based on the significant decrease in the error rate. Statistical significance was set at *p*  <  0.05 in the used tests.

## 3. Results

### 3.1. V. vulnificus, V. cholerae, and V. alginolyticus on Beaches

The *vvhA* gene of *V. vulnificus* was found in 54.2% of tested samples and was present at all investigated beaches during different months, except for Melnragė beach in September. The prVC gene (*V. cholerae*) was found on all beaches (in 37.5% of samples). In July, three targeted *Vibrio* species were detected (Supplementary Table S2).

**Figure 2:**
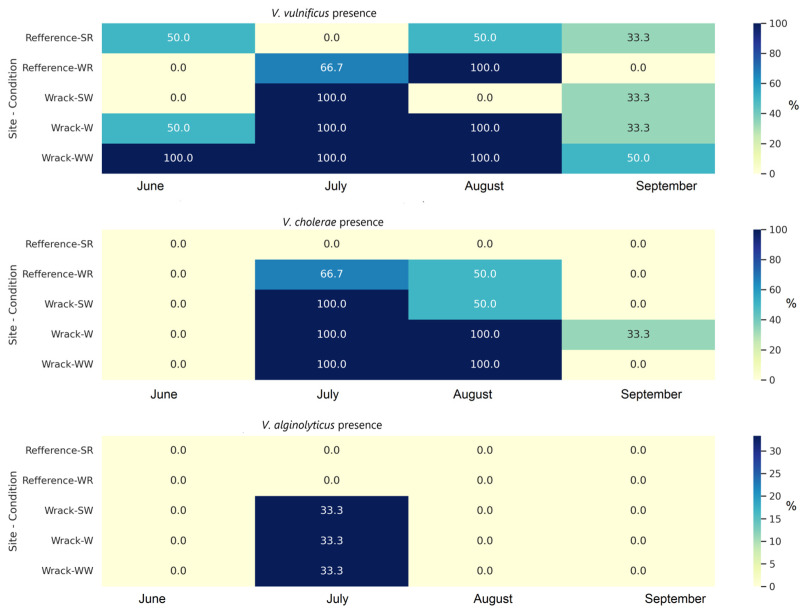
The identification frequency of *Vibrio* species (% from analyzed samples) by PCR during different months in environmental samples (SR—sand in reference; WR—water in reference; SW—sand in wrack; W—wrack; WW—water in wrack).

In water at wrack accumulation sites and within the wrack itself, *V. vulnificus* was present in 88.9% and 70% of the samples, respectively. *V. vulnificus* appeared in only 30% of the sand samples from reference sites. *V. cholerae* in water at wrack accumulation sites and in the wrack was found in 55.6% and 60% of samples, respectively, and it was not detected in sand from the reference site. *V. alginolyticus* was detected only once in July and at the wrack accumulation site (Figure 2). *V. parahemolyticus* was not detected during this research.

**Figure 3:**
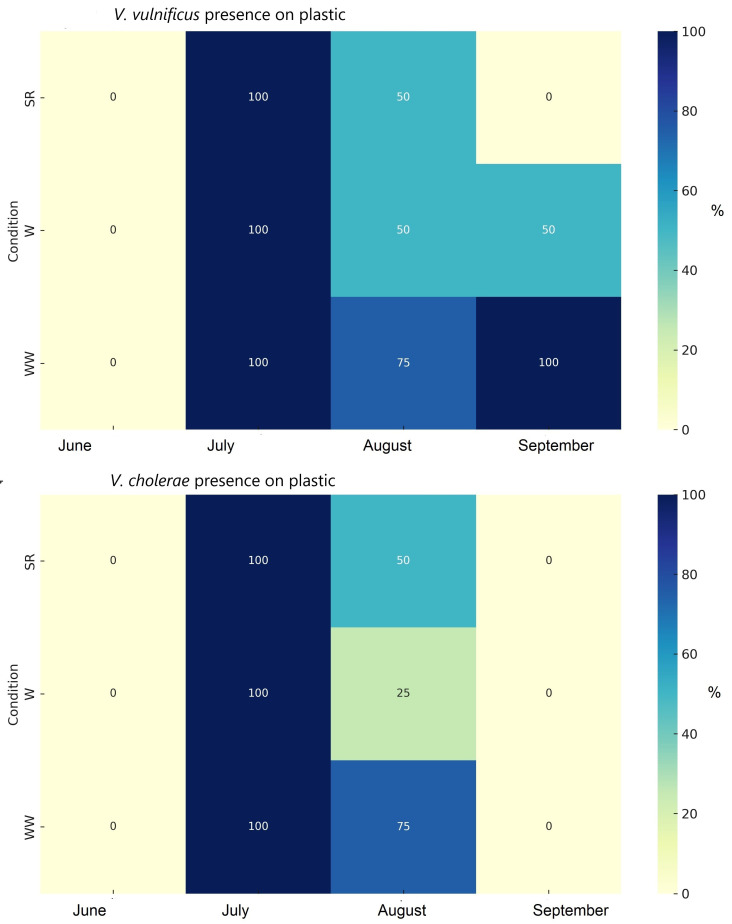
The identification frequency of *Vibrio* species (% from analyzed plastic samples) by PCR on plastic samples during different months (SR—sand in reference; W—wrack; WW—water in wrack).

*V. vulnificus* and *V*. *cholerae* were found in 53.3% and 40% of the analyzed plastic samples, respectively. *V. vulnificus* was found in 50% of the plastic samples from the wrack, 66.7% of the samples from the water with wrack, and 44.4% of the samples from the reference water. The highest detection rates for *V. vulnificus* and *V. cholerae* on plastic were in July (73%) and August (70% and 60%, respectively). *V. cholerae* was found in 30% of the plastic samples from the wrack, in 44.4% of the samples from water in the wrack, and from the reference site (Figure 3).

The chi-square test revealed that both *Vibrio* species in the environment and on plastic were significantly associated with the month. The environmental parameters, such as temperature, oxygen, and turbidity, significantly differed among months (Supplementary Table S4). Additionally, *V. cholerae* was significantly linked to the subsite (wrack or reference), while the same bacteria attached to plastics were associated with the beach location (Supplementary Table S3). 

The highest concentrations of the *V. vulnificus vvhA* gene were detected in Karklė and Palanga in July, specifically in water containing wrack (2.6 × 10^7^ and 2.3 × 10^7^ GC/100 mL, respectively). In the same month, the highest concentration of *V. vulnificus* in reference water (1.0 × 10^6^ GC/100 mL) was found in Palanga (Supplementary Table S2).

**Figure 4:**
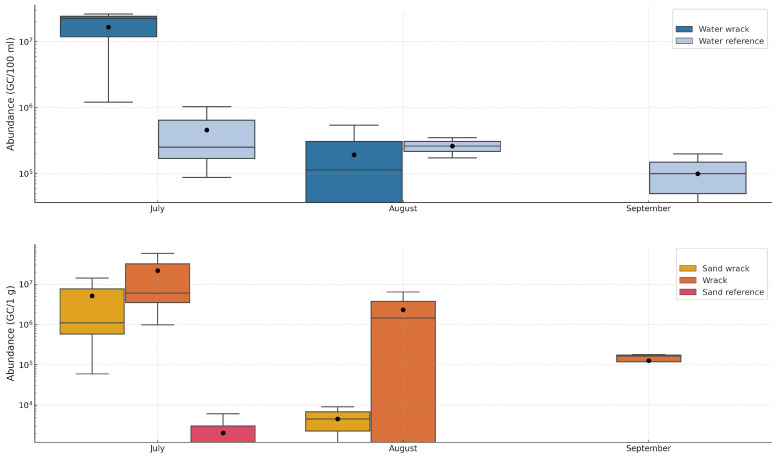
The average abundances of *V. vulnificus* (*vvhA* gene) under various conditions in July, August, and September are shown, with whiskers indicating the maximum and minimum values. The line within each box plot represents the median, and the circles denote the average quantities.

There were significant differences in *V. vulnificus* abundance among months, both in water and sand with wrack, when, on average, the highest quantity was found in July (Kruskal–Wallis = 8.824 and 7.875, respectively; *p* < 0.05, n = 9) (Figure 4). There were no significant differences in abundance between months in other conditions. In July, a higher average abundance was observed in the water with wrack (1.7 ± 1.34 × 10^7^ GC/100 mL) compared with the reference conditions (4.6 ± 5.05 × 10^5^ GC/100 mL). However, it was not statistically significant (*p* > 0.05).

Spearman correlation coefficients calculated between *V*. *vulnificus* and water parameters were significant: positive with temperature, SPM, chl-a, and turbidity and negative with oxygen (Table 1). In water with wrack, *V. vulnificus* significantly correlated with diatoms + dinoflagellates, chl-a concentration, and the proportion of *Vertebrate fucoides* in the wrack, and negatively with oxygen.

**Figure 5:**
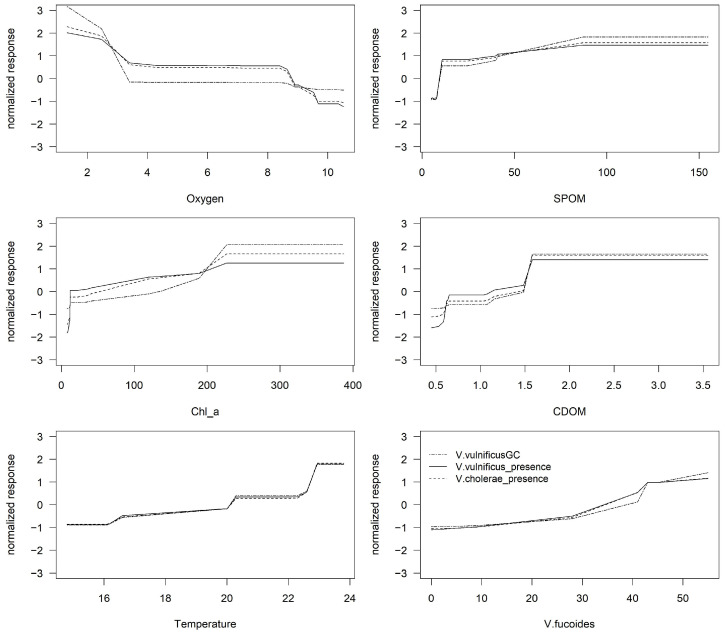
Partial dependence plots of environmental parameters on *V. vulnificus* abundance and presence and *V. cholerae* presence using MRF.

The MRF model with the environmental factors explained 21% of the variation in the abundance and presence of *V*. *vulnificus* and the presence of *V*. *cholerae* (Figure 5).

For *V*. *vulnificus* abundance, the most important factors were the coverage of *V*. *fucoides,* chl-a, and oxygen; for *V*. *vulnificus* presence, they were chl-a and CDOM; and for the presence of *V. cholerae*, they were temperature, chl-a, and oxygen (Supplementary Table S5).

### 3.2. Vibrio Diversity Based on 16S rRNA Sequencing on Šventoji Beach During Single- and Multiple-Day Sampling

During the overall studied period, 14 different OTUs belonging to the *Vibrio* genus were identified (11 in 2021 and 9 in 2022). Some OTUs showed high similarity (≥97%) to specific *Vibrio* taxa, such as *V. ostreae*, *V. cholerae*, *V. anguillarum*, *V. pommerensis*, *V. rumoiensis*, *Vibrio sp. MI-15*, and *Vibrio sp. F74* (Supplementary Figure S1).

**Figure 6:**
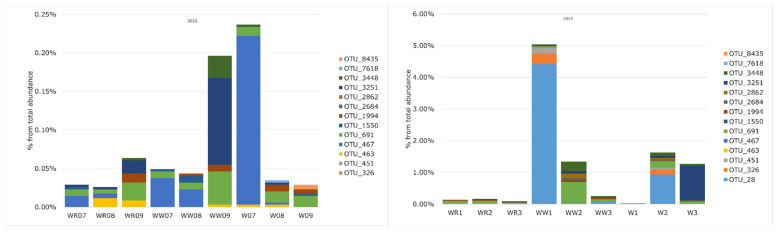
The relative abundances of OTUs assigned to *Vibrio* in different conditions (WR—water in reference site; WW—water in wrack site; W—wrack) during single-day sampling events in 2021 (7—July; 8—August; 9—September) and during multiple-day events in 2022 (1—first day of sampling; 2—second day; 3—fourth day).

During the single-day sampling in 2021, OTU 691 (*V. anguillarum*) was found in all samples. A higher relative abundance of *Vibrio* OTUs was observed in wrack sampled in July, dominated by OTU 467 (*V*. *cholerae*), and in water with wrack in September, dominated by OTU 3251 (*V*. *ostreae*) (Figure 6). In other environments during June, July, and August, the relative abundance of *Vibrio* OTUs was similar.

**Figure 7:**
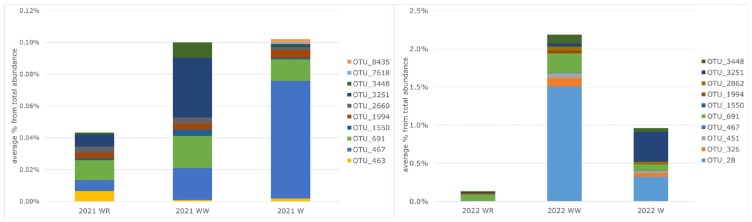
The average relative abundances of OTUs assigned to *Vibrio* in different conditions (WR—water in reference site; WW—water in wrack site; W—wrack) during single-day sampling events in 2021 and multiple-day sampling in 2022.

In 2021, 8 *Vibrio* OTUs in water without and with wrack were identified, while 10 were identified in wrack. On average, a higher abundance of *Vibrio* OTUs was observed in wrack or water with wrack (Figure 7). In wrack, the highest abundance was of OTU 467 (*V*. *cholerae*), while in water with wrack, it was of OTU 3251 (*V*. *ostreae*).

During multi-day sampling in 2022, 8 *Vibrio* OTUs in water without wrack and 10 OTUs in wrack and water with wrack were identified. OTU 3448 (*V. pommarensis*) was found in all samples. High abundance (up to 5% of total abundance) was observed in water with wrack on the first accumulation day, dominated by OTU 28. Comparably higher abundance (>1%) was identified in water with wrack (dominated by OTU 691 (*V. anguillarum*)) on the second day and in wrack on the second (OTU 28) and fourth (OTU 3251) days. The abundance of *Vibrio* OTUs was similar in water from the reference site during the studied period. On average, higher abundance was observed in water with wrack accumulation, and the lowest abundance was observed in reference water (Figure 7).

Some OTUs were found only in wrack and water with wrack: in 2021, these were OTU 7618 and OTU 8435, and in 2022, they were OTU 326 and OTU 451 (*Vibrio* sp. F74).

Hierarchical cluster analysis based on the Bray–Curtis dissimilarity group did not show a statistically significant difference between the sites (ANOSIM Global R = −0.02634, *p* = 0.571). However, samples from July had a similar composition of *Vibrio* OTUs (Supplementary Figure S2).

Several environmental parameters showed significant correlations (*p* < 0.05) with the relative abundance of specific OTUs. Temperature was negatively correlated with the abundance of OTU 463, while salinity was negatively correlated with OTU 467 (*V. cholerae*). Cryptophytes exhibited significant positive or negative correlations with nearly all OTUs, with varying degrees (Table 2). Additionally, a higher proportion of *Vertebrata fucoides* and *Ulva intestinalis* in the wrack was positively correlated with OTUs 28 and 326.

## 4. Discussion

The presence of Vibrio species such as *V. vulnificus*, *V. fluvialis*, *V. anguillarum*, *V. cholerae*, *V. alginolyticus*, *V. cincinnatiensis*, *V. furnissi*, *V. navarrensis*, *V. harveyi*, and *V. mentschinkowi* that are known for causing infections [41,42,43,44,45,46] has recently been found in coastal waters of the SE Baltic Sea [7,33]. Moreover, the best predictors of *V*. *vulnificus* along the Baltic Sea’s salinity gradients were eutrophication-related parameters, such as particulate organic carbon, nitrogen, phosphate, and the occurrence of potential phytoplankton blooms [8]. 

Along with phytoplankton blooms, macroalgae wrack accumulations are observed after stormy conditions on coastal beaches of the Baltic Sea, negatively affecting water quality [47] and enriching the coastal ecosystems with nutrients. Due to eutrophication, the contribution of ephemeral and nutrient-opportunistic seaweeds is increasing in the wrack of the Baltic Sea, thus increasing nuisance compared to late successional macrophytes [48]. The wrack accumulated on the coast favors the survival and proliferation of bacteria and pathogens related to fecal pollution [13,24]. However, based on our systematic review analysis, we found only one study on *Vibrio* species from the polluted Mediterranean Sea [49], where the microbiome of *Posidonia* wrack was analyzed. In our case, the wrack consisted of perennial macroalgae such as *Furcellaria lumbricalis* and *Vertebrata fucoides* (relative abundance varied from 48 to 81%) and ephemeral algae such as *Cladophora* (relative abundance varied from 3 to 28%) [21].

Our study revealed that during the recreational period, at least three species of *Vibrio* were identified or quantified using species-specific primers in the wrack accumulation sites along the Baltic Sea: *V*. *vulnificus*, *V*. *cholerae*, and *V. alginolyticus*. Up to 14 OTUs belonging to the *Vibrio* genus were identified in a wrack-affected environment based on 16s rRNA gene amplicon sequencing. *V. cholerae* was identified in samples using both approaches. However, caution should be exercised when identifying species within the genus *Vibrio* based only on a fragment of the 16S rRNA gene, as this marker lacks the necessary phylogenetic resolution for precise species determination [50,51].

*V*. *vulnificus* and *V*. *cholerae* were more frequently identified in wrack environments than in sites without wrack accumulation. *V. alginolyticus*, identified on the Lithuanian coast for the first time, was found only in environments with wrack accumulation. Some OTUs (OTU 7618, 8435, OTU 326, and OTU 451 (*Vibrio* sp. F74)) were found only in wrack accumulation sites, indicating that they might be either constituents of live macroalgae or taking part in the degradation process after macroalgae accumulate. Kolda et al. [49] found that *Vibrio* spp. in *Posidonia oceanica*-dominated wrack contributed not only to fermentation and aerobic chemoheterotrophy but also to nitrate reduction and associations with animal parasites. For example, in our study, the relative abundance of OTU 326 significantly correlated with the presence of *Vertebrata fucoides* or *Ulva intestinalis.* Some *Vibrio* species are known for possessing algae-specific polysaccharidases (e.g., agarases, carrageenases, and alginate lyases) [52] and participating in macroalgae degradation. For example, *V. alginolyticus* can degrade agar, cellulose, sodium alginate, xylan, laminarin, and carrageenan [53]. On the other hand, macroalgae polysaccharides or live-macroalgae-associated bacteria are known to inhibit the growth of potentially pathogenic bacteria such as *V*. *anguillarum*, *V*. *cholerae*, and others [54,55]. However, as macroalgae degrade, associated microorganisms may lose the ability to defend against pathogenic microorganisms. The degradation of tissue leads to the release of nutrients [25], which could support an increase in microorganisms capable of feeding on the released nutrients. Some of them might be opportunistic pathogens. Our study limitation was that we did not analyze the nutrients (such as nitrogen or phosphorus); thus, we can only hypothesize about their significance, as demonstrated in other studies [8,9]. Particulate organic carbon and nitrogen were closely associated with chl-a in research on *V. vulnificus* along the salinity gradient in the Baltic Sea [8]. In our case, chl-a concentrations were significantly higher in water with wrack accumulations than in the reference. The nutrients released from the degrading wrack might affect the growth of phytoplankton, including algae that produce chlorophyll-a. Chl-a was an important environmental parameter explaining the abundance and occurrence of *V. vulnificus* and *V. cholerae* in our case. The importance of chl-a for *V*. *vulnificus* abundance was demonstrated in other studies as well. Genetic markers of *V*. *vulnificus* were observed when chl-a concentrations ranged from 5 to 25 μg/L in the Chesapeake Bay [56], and a correlation with chl-a was also found in the Baltic Sea [8].

As another important variable, the water temperature was related to the higher detection frequency and abundance of *V. vulnificus* in July, when it reached 23.8 °C [21]. Numerous authors have shown temperature to be the main driving factor of *Vibrio* abundance in the Baltic Sea, especially for potentially pathogenic *Vibrio* species [8,9]. Salinity, as one of the environmental factors that influence the geographic distribution of *Vibrio* species and affect the concentration of certain *Vibrio* species [7,8], was not significant for *V. vulnificus* abundance or presence in our case. However, the highest abundances of *V. vulnificus* were observed on the Palanga and Karklė beaches, where wrack accumulation occurred under salinity conditions of around 6 PSU [21]. Based on 16S rRNA gene amplicon sequencing at Šventoji beach, where salinity ranged from 0.2 PSU to 6.6 PSU during our sampling, we found that the relative abundance of OTU 467 (clade *V*. *cholerae*) increased as salinity decreased. On this beach, wrack accumulated near the Šventoji river, whose outflow diluted the saline conditions [22], potentially contributing to the shift in *Vibrio* composition. *V. cholerae* in the Baltic Sea was found across a salinity gradient ranging from 0.24 to 29.4 PSU [7]. However, previous studies on the Lithuanian coast reported higher abundances of *V. cholerae* in the low-salinity or freshwater conditions of the Curonian Lagoon [2], compared to the higher-salinity conditions of the Baltic Sea.

*Vibrio* is an early colonizer of plastics, and higher nutrients could enhance the faster establishment of *Vibrio* populations in the Baltic Sea [50]. In our case, *V. vulnificus* and *V. cholerae* were identified on all studied plastic samples from July, with a higher frequency on plastic from water with wrack accumulations; however, they were also found on plastic from the coast without wrack accumulations. In the reference sites, the higher frequency of *Vibrio* on plastic in the sand, compared to its presence in the sand, was most probably due to biofilm formation that develops in the aquatic environment on the plastic and later, when the plastic is deposited on the coast, can protect *V. vulnificus* from environmental stressors and enhance its survival [19,57]. However, more studies or simulations could be performed to understand this process.

OTU 691 (clade *V. anguillarum*) was found in all samples and increased in abundance during multi-day sampling on the second day of wrack accumulation in water. That day, higher average chlorophyll and blue-green algae abundances were observed compared to other sampling days (67.3 and 37.15 mg m^−3^, respectively) [21]. *V. anguillarum* is abundant in the brackish waters of the Baltic Sea and is known to correlate with chlorophyll and cyanobacterial abundance [58]. Also, it is known as a fish pathogen, capable of infecting other marine animals and, in some rare cases, humans [41,59]. On the other hand, this species is a seaweed-associated microorganism that positively affects the settlement of zoospores of *Enteromorpha* or *Ulva* [12]. OTU 3448 found in both years showed high similarity to *Vibrio navarrensis* biotype *pommerensis* that was first isolated from the Baltic Sea [60] and is known for causing human infections [46] and able to utilize lactose as a sole carbon source, which is also specific to *V. vulnificus*. In our study, there was a higher relative abundance of this OTU in water in wrack both in September of 2021 and on the second day of sampling in 2022.

Grazing pressures on cyanobacteria in the environment could explain different OTUs’ correlation with the abundance of cryptophytes. Riedinger et al. [8] found that when there is a higher abundance of cryptophyte *Teleaulax*, there is a decrease in certain cyanobacteria, nutrients, and *V. vulnificus* abundance. This may be attributed to the potential role of cryptophytes due to the grazing of cyanobacteria, limiting *Vibrio* access to the related organic nutrients [8]. The inconsistent composition of *Vibrio* during the studied period, related to the presence or absence of accumulated wrack, could be attributed to the sampling strategy based on the single-day sampling campaigns (except in 2022) when the samples were taken at different degradation stages. For example, following the allocation of samples from September 2021 with samples of the fourth sampling day in 2022 in a dendrogram, we can assume that the wrack was at a similar succession stage. However, a different research strategy should be used to prove this, considering all wrack succession stages and using more frequent sampling. Using only molecular methodologies can also be considered as our study limitation. 16s gene amplicon sequencing allows the detection of *Vibrio* present in a non-culturable state and less-characterized *Vibrio* species compared to traditional culturing methods. However, this method cannot differentiate between live and dead cells, limiting its ability to assess the viability of potentially pathogenic *Vibrio*; moreover, depending on the sequencing depth, it can limit the detection of low-abundance species. In contrast, culturing on selective media can provide information on viable *Vibrio*, and isolates can be used for further characterization. Recent advancements in qPCR can supplement the assessment of live cells and non-viable cell quantification of dead cells [61].

## 5. Conclusions

Accumulated algal wrack on coastal sandy beaches plays an important role due to habitat and nutrient provision; however, it might be an issue for beaches used for recreational activities. As our study revealed, in such wrack accumulations, there are potentially pathogenic *Vibrio* present, with their abundances increasing during periods of higher water temperature, which is usually related to increased beachgoer numbers. *Vibrio* presence should be considered when managing wrack accumulations on recreational beaches to prevent people from being exposed to potential pathogens. From the ecological perspective, more research should be conducted to analyze how, in such ecosystems, the microbial food web changes during wrack degradation stages, including the nutrient release and grazing effect, and what implications it has on the abundance and presence of potentially pathogenic *Vibrio* species.

## Figures and Tables

**Table 1:** Spearman correlation of *V. vulnificus* with environmental parameters in water samples based on qPCR results (statistically significant correlation coefficients are marked by * (*p* < 0.05) or ** (*p* < 0.01)).

	*V*. *vulnificus*, Gene Copies/100 mL in All Sites (N = 18)	*V*. *vulnificus*, Gene Copies/100 mL in Wrack Sites (N = 9)	*V*. *vulnificus*, Gene Copies/100 mL in Reference Sites (N = 9)
Temperature,°C	0.549 *	0.58	0.43
Oxygen, mg L^−1^	−0.651 **	−0.74 *	−0.68 *
Salinity, PSU	−0.083	−0.026	0.40
pH	−0.332	−0.65	−0.31
SPM, g m^−3^	0.491 *	0.43	0.57
SPIM, g m^−3^	0.523	0.49	0.63
SPOM, g m^−3^	0.368	0.38	0.22
CDOM, m^−1^	0.363	0.56	0.31
Blue-green algae, mg chl-a m^−3^	0.099	0.13	−0.20
Diatoms + dinoflagellates, mg chl-a m^−3^	0.371	0.76 *	0.30
Green algae, mg chl-a m^−3^	−0.033	−0.08	−0.17
Cryptophytes, mg chl-a m^−3^	0.0159	0.313	−0.62
Chl-a concentration, mg m^−3^	0.481 *	0.78 *	0.68 *
Turbidity, NTU	0.549 *	0.58	0.43
*Furcellaria lumbricalis*, %	−0.070	−0.64	
*Vertebrata fucoides*, %	0.379	0.79 *	
*Cladophora rupestris*, %	0.372	0.61	
*Cladophora glomerata*, %	−0.152	−0.13	

**Table 2:** Spearman correlation heatmap between OTU relative abundances and environmental parameters in the water from Šventoji beach. Only significantly important correlation coefficients (*p* > 0.05) are provided. The color gradient indicates the strength and direction of the correlations: blue cells indicate negative correlations, and red cells represent positive correlations.

	OTU_28	OTU_326	OTU_451	OTU_463	OTU_467	OTU_691	OTU_1550	OTU_1994	OTU_2684	OTU_2862	OTU_3251	OTU_3448
Temperature				−0.66								
Salinity					−0.74						0.58	
Chl-a												
Green algae												
Blue-green algae	0.69	0.67		−0.59						0.73		
Diatoms + dino algae												
Cryptophytes	−0.76	−0.64	−0.18	0.32	0.40	−0.62	0.66	−0.64		−0.86		−0.61
*Furcelaria lumbricalis*												
*Vertebrata fucoides*		0.70										
*Ulva intestinalis*	0.63	0.72										
*Cladophora* sp.												

## Data Availability

The original contributions presented in the study are included in the article/Supplementary Material, further inquiries can be directed to the corresponding author.

## Supplementary Materials

The following supporting information can be downloaded at https://www.mdpi.com/article/10.3390/microorganisms12102101/s1: Table S1: Targeted bacterial groups and oligonucleotide primers used for qPCR and PCR; Table S2: Average quantities of *V. vulnificus* and presence of *Vibrio* based on PCR results (W—wrack accumulation area; R—reference area with no wrack); Table S3: The presence of *V. vulnificus*, *V. cholerae*, and *V. alginolyticus* in environmental samples and on plastic. Numbers in bold refer to significant values; Table S4: Kruskal–Wallis test results for water environmental parameter differences by month, site, and condition. Statistically significant (*p* < 0.05) differences are marked by *; Table S5: The relative importance of environmental factors in water for response variables: *V*. *vulnificus* abundance, *V*. *vulnificus*, and *V*. *cholerae* presence based on the MRF model. Table S6. The information related to systematic literature review data. Figure S1: Phylogenetic tree of all *Vibrio* OTUs from the water of the Šventoji beach. Two species, *Photobacterium* and *Aeromonas*, were used as outgroups. The tree was reconstructed by using the neighbor-joining method. Bootstrap values (>50%) are shown at the nodes; Figure S2. Cluster diagram of Bray–Curtis similarities calculated from square-root-transformed relative OTU abundances for each sample (06—June; 07—July; 08—August; 09—September; 1—first day; 2—second day; 3—the fourth day; SR—sand in reference; WR—water in reference; SW—sand in the wrack; W—wrack; WW—water in water wrack). References [62,63,64,65] are cited in the Supplementary Materials file.
