# Supplementary material for: Potentially Pathogenic Vibrio spp. in Algal Wrack Accumulations on Baltic Sea Sandy Beaches
Source: Microorganisms. 2024 Oct 21;12(10):2101. doi: 10.3390/microorganisms12102101 (PMC11509979; doi:10.3390/microorganisms12102101)
Supplement: Supplementary file 1 [file microorganisms-12-02101-s001.zip › microorganisms-3209085-supplementary.pdf]

Supplementary Table S1. Targeted bacterial groups and oligonucleotide primers used for PCR and qPCR

Assay	Targeted bacterial group	Primers	Reference
Conventional PCR			
vvha_785[1]<br>vvhA_1303[1]	*V. vulnificus*	F: 5'-CCGCGGTACAGGTTGGCGCA-3'<br>R: 5'-CGCCACCCACTTTCGGGCC-3'	(Kaysner and DePaola, 2001)
prVC[2]	*V. cholerae*	F: 5'-TTAAGCSTTTTCRCTGAGAATG-3'<br>R: 5'-AGTCACTTAACCATACAACCCG-3'	(Chun et al., 1999)
toxR	*V. parahaemolyticus*	F: 5′-GTCTTCTGACGCAATCGTTG-3′<br>R: 5′-ATACGAGTGGTTGCTGTCATG-3′	(Kim et al., 1999)
gyrB	*V. alginolyticus*	F: 5’-TCAGAGAAAGTTGAGCTAACGATT-3’<br>R: 5’-CATCGTCGCCTGAAGTCGCTGT-3’	(Luo and Hu, 2008)
Real-time PCR			
vvha	*V. vulnificus*	F: 5′-TGTTTATGGTGAGAACGGTGACA-3′<br>R: 5′-TTCTTTATCTAGGCCCCAAACTTG-3′<br>Probe: (FAM)-CCGTTAACCGAACCACCCGCAA-(TAMRA)	(Campbell and Wright, 2003)

[1]vvhA is with virulence-associated gene encoding hemolysin

[2]amplifies a portion of the intergenic spacer region between the 16S and 23S rRNA genes and is specific for *V. cholerae*

Supplementary Table S2. Average quantities of *V. vulnificus* based on qPCR and presence of *V. vulnificus, V. cholerae* and *V. alginolyticus* based on PCR results (W - wrack accumulation area, R - reference area with no wrack)

Parameter	W/R	Melnragė				Karklė		Palanga	Šventoji		
		06.02	06.18	08.10	09.16	07.27	09.17	07.29	07.07	08.27	09.17
**WATER**											
V. vulnificus, gene copies/100 mL	W	-	-	541168	-	26160146	N.D.[1]	22369911	1212553	227878	198755
V. vulnificus, presence	W	+	+	+	-	+	N.D.[1]	+	+	+	+
V. cholerae, presence	W	-	-	+	-	+	-	+	+	+	-
V. alginolyticus, presence	W	-	-	-	-	-	-	+	-	-	-
V. vulnificus, gene copies/100 mL	R	-	-	173209	-	251246	N.D.[1]	1032721	87444	349965	-
V. vulnificus, presence	R	-	-	+	-	+	N.D.[1]	+	-	+	-
V. cholerae, presence	R	-	-	+	-	+	-	+	-	-	-
V. alginolyticus, presence	R	-	-		-	-	-	-	-	-	-
**SAND**											
V. vulnificus, gene copies/1 g	W	-	-	9023	-	14203479	-	1092394	59420	-	-
V. vulnificus, presence	W	-	-	-	-	+	-	+	+	-	+
V. cholerae, presence	W	-	-	+	-	+	-	+	+	-	-
V. alginolyticus, presence	W	-	-	-	-	-	-	+	-	-	-
V. vulnificus, gene copies/1 g	R	-	-	-	-	-	-	6085	-	-	-
V. vulnificus, presence	R	-	+	-	-	-	-	-	-	+	+
V. cholerae, presence	R	-	-	-	-	-	-	-	-	-	-
V. alginolyticus, presence	R	-	-	-	-	-	-	-	-	-	-
**WRACK**											
V. vulnificus, gene copies/1 g		-	-	2883297	171236	6003993	179134	58122938	975460	6395924	157328
V. vulnificus, presence		-	+	+	+	+	-	+	+	+	+
V. cholerae, presence		-	-	+	+	+	-	+	+	+	-
V. alginolyticus, presence		-	-	-	-	-	-	+	-	-	-

[1] - no data, samples were not collected due to the absence of wrack

Supplementary Table S3. The presence of *V. vulnificus*, *V. cholerae*, and *V. alginolyticus* in environmental samples and on plastic. Statistically significant differences are marked by *(p<0.05) or ** (p<0.01)

Species		**Month** June, July, August, September		**Beach** Melnrage, Karkle, Palanga, Sventoji		**Subsite** Wrack, Reference		**Condition** Water wrack, sand wrack, wrack, water reference, sand reference	
		Chi2	P-Value	Chi2	P-Value	Chi2	P-Value	Chi2	P-Value
V. vulnificus	Environment	17.6**	0.007	10.0	0.12	4.0	0.14	13.7	0.090
V. cholerae		33.7**	0.000	10.0	0.12	6.5*	0.04	14.0	0.081
V. alginolyticus		18.3**	0.005	35.6**	0.00	2.2	0.33	7.5	0.482
V. vulnificus	Plastic	10.24*	0.016	3.4	0.18	0.06	0.8	0.973	0.614
V. cholerae		15.42**	0.001	9.2**	0.001	0	1	0.56	0.75

Supplementary Table S4. Kruskal-Wallis test results for water environmental parameter differences by month, site, and condition. Statistically significant (p<0.05) differences are marked by *

	Month (June, July, August, September)		Beach (Melnragė, Karklė, Palanga, Šventoji)		Site (Wrack, Reference)	
Parameter	H-value	p-value	H-value	p-value	H-value	p-value
Temperature, °C	11.65*	0.01	0.27	0.61	0.00	0.96
Oxygen, mg l^{-1}	7.99*	0.05	0.42	0.52	5.07*	0.02
Salinity, PSU	2.03	0.57	0.07	0.80	0.44	0.51
pH	2.14	0.54	2.40	0.12	5.48*	0.02
SPM, g m^{-3}	2.83	0.42	2.41	0.12	8.25*	0.00
SPIM, g m-3	3.45	0.33	2.41	0.12	8.25*	0.00

SPOM, g m^{-3}	1.60	0.66	0.60	0.44	10.41*	0.00
CDOM, m^{-1}	2.07	0.56	0.15	0.70	9.30*	0.00
Blue-green algae, mg chl-a m^{-3}	0.57	0.90	2.21	0.14	2.53	0.11
Diatoms+Dino algae, mg chl-a m^{-3}	0.37	0.95	0.02	0.90	8.24*	0.00
Green algae, mg chl-a m^{-3}	1.88	0.60	0.42	0.52	0.33	0.57
Cryptophytes, mg chl-a m^{-3}	0.12	0.99	1.35	0.25	5.48*	0.02
Chl-a concentration, mg m^{-3}	1.15	0.77	0.15	0.70	12.79*	0.00
Turbidity, NTU	11.65*	0.01	0.27	0.61	0.00	0.96

Supplementary Table S5. The relative importance of environmental factors in water for response variables: *V. vulnificus* abundance, *V. vulnificus* and *V. cholerae* presence based on the MRF model

**Environmental parameter**	*V. vulnificus,* GC	*V. vulnificus,* presence	*V. cholerae,* presence
Temperature, °C	< 0	0.18	1
Oxygen, mg l^{-1}	0.63	0.04	0.64
Salinity, PSU	<0	<0	<0
Blue-green algae, mg chl-a m^{-3}	<0	0.09	0.08
Chl-a concentration, mg m^{-3}	0.60	1.00	0.96
CDOM, m^{-1}	0.33	0.39	0.36
SPOM, g m^{-3}	0.31	0.15	0.06
F. lumbricalis	<0	0.05	<0
V. fucoides	1.00	0.14	0.48

Supplementary Table S6. The information related to systematic literature review data.

Link	Title	Notes	Year	Freshwater/ marine ecosystem	Sampling site	Type of research	What was researched	Methods (bacteria species identification and quantification)	Macroalgae species	Vibrio species identified	Environmental parameters analysed	Result
Beach Wracks Microbiome and its Putative Function in Plastic Polluted Mediterranean Marine Ecosystem by Anamarija Kolda, Maja Mucko, Ana Rapljenović, Zrinka Ljubešić, Kristina Pikelj, Željko Kwokal, Hana Fajković, Vlado Cuculić :: SSRN	Beach wracks microbiome and its putative function in plastic polluted Mediterranean marine ecosystem	A preprint	2024	Marine	Adriatic Sea, Croatia	In situ sampling	Wrack, plastic, microbial composition	Identification: 16s rRNA sequencing (OTU)	Two communties of seagrass: Cymodocea-Zostera and P. oceanica	*Vibrio* sp., *V. parahaemolyticus*, *V. natriegens*, *V. halioticoli*, *V. pomeroyi*, *V. rumoiensis*, *V. harveyi*	Sand grain size, carbonate content of sand, macrophyte species in wrack, plastic type, beach type (sheltered or exposed)	There was significant dissimilarity between sample groups with *Vibrio*, Cobetia and Planococcus shaping the “Exposed” beach sample group and Cyclobacteriaceae and Flavobacterium shaping the “Sheltered” beach sample group

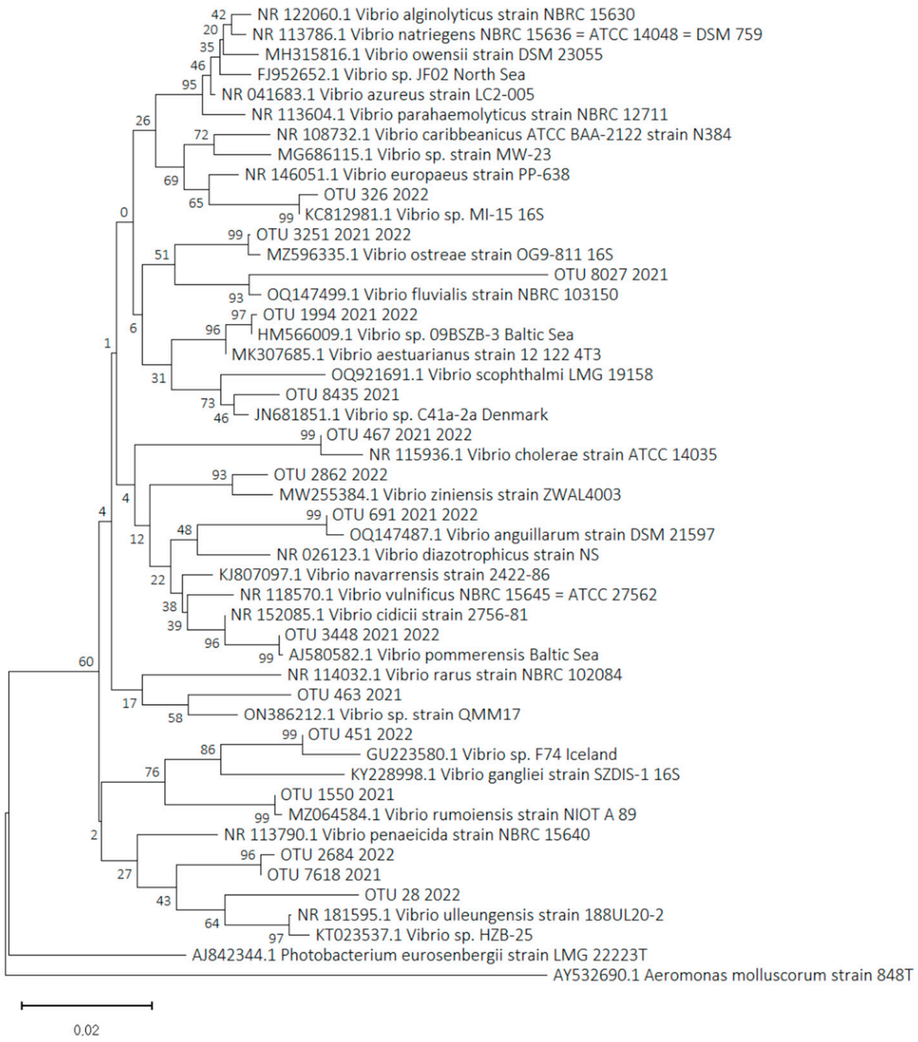

Supplementary Figure S1: Phylogenetic tree of all *Vibrio* OTUs from water of the Šventoji beach. Two species, *Photobacterium* and *Aeromonas*, were used as outgroups. Tree was reconstructed by using the neighbour-joining method. Bootstrap values (>50%) are shown at the nodes.

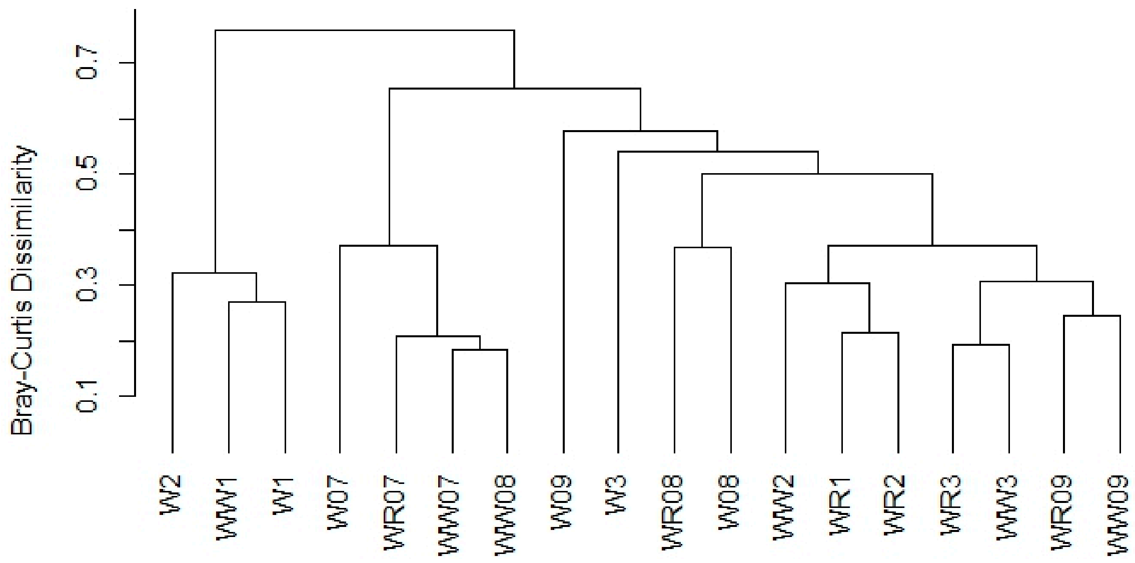

Supplementary Figure S2. Cluster diagram of Bray-Curtis similarities calculated from square-root transformed relative OTU abundances of *Vibrio* for each sample. (07 – July, 08 – August, 09 – September, 1 - first day, 2 - second day, 3 - the fourth day, SR – sand in reference, WR - water in reference, SW - sand in the wrack, W – wrack, WW – water in water wrack).

Literature:

Kaysner, C.A. and DePaola, A. (2001) Vibrio. In Bacteriological Analytical Manual Online, Chapter 9. Rockville, MD: US Food and Drug Administration.

Campbell, M.S. and Wright, A.C., 2003. Real-time PCR analysis of Vibrio vulnificus from oysters. *Applied and environmental microbiology*, *69*(12), pp.7137-7144.

Kim, Y.B., Okuda, J.U.N., Matsumoto, C., Takahashi, N., Hashimoto, S. and Nishibuchi, M., 1999. Identification of Vibrio parahaemolyticus strains at the species level by PCR targeted to the toxR gene. *Journal of Clinical Microbiology*, *37*(4), pp.1173-1177.

Chun, J., Huq, A. and Colwell, R.R., 1999. Analysis of 16S-23S rRNA intergenic spacer regions of *Vibrio* cholerae and Vibrio mimicus. *Applied and Environmental Microbiology*, *65*(5), pp.2202-2208.
